# Epithelial Sodium Channel-Mediated Sodium Transport Is Not Dependent on the Membrane-Bound Serine Protease CAP2/Tmprss4

**DOI:** 10.1371/journal.pone.0135224

**Published:** 2015-08-26

**Authors:** Anna Keppner, Ditte Andreasen, Anne-Marie Mérillat, Julie Bapst, Camille Ansermet, Qing Wang, Marc Maillard, Sumedha Malsure, Antoine Nobile, Edith Hummler

**Affiliations:** 1 Department of Pharmacology & Toxicology, University of Lausanne, Lausanne, Switzerland; 2 Department of Medicine/Division of Nephrology and Hypertension, Lausanne University Hospital (CHUV), Lausanne, Switzerland; 3 Department of Medicine/Physiology, University of Fribourg, Fribourg, Switzerland; 4 Institut Universitaire de Pathologie, Lausanne University Hospital (CHUV), Lausanne, Switzerland; Nagoya University, JAPAN

## Abstract

The membrane-bound serine protease CAP2/Tmprss4 has been previously identified *in vitro* as a positive regulator of the epithelial sodium channel (ENaC). To study its *in vivo* implication in ENaC-mediated sodium absorption, we generated a knockout mouse model for CAP2/Tmprss4. Mice deficient in CAP2/Tmprss4 were viable, fertile, and did not show any obvious histological abnormalities. Unexpectedly, when challenged with sodium-deficient diet, these mice did not develop any impairment in renal sodium handling as evidenced by normal plasma and urinary sodium and potassium electrolytes, as well as normal aldosterone levels. Despite minor alterations in ENaC mRNA expression, we found no evidence for altered proteolytic cleavage of ENaC subunits. In consequence, ENaC activity, as monitored by the amiloride-sensitive rectal potential difference (ΔPD), was not altered even under dietary sodium restriction. In summary, ENaC-mediated sodium balance is not affected by lack of CAP2/Tmprss4 expression and thus, does not seem to directly control ENaC expression and activity *in vivo*.

## Introduction

The regulation of sodium balance throughout the body is important to maintain blood volume and blood pressure. In tight epithelia as in kidney and colon, aldosterone promotes sodium reabsorption through the amiloride-sensitive epithelial sodium channel ENaC [1]. This channel was initially identified in the colon of rats challenged with a low salt diet [2,3]. ENaC is composed of three subunits, Scnn1a, Scnn1b, and Scnn1g, sharing 30% homology with each other at the protein level [3].

One regulatory mechanism of ENaC-mediated sodium reabsorption is achieved through channel-activating proteases (CAPs) as e.g. CAP1 (Prss8 or prostasin), CAP2 (Tmprss4) and CAP3 (ST-14 or matriptase) [4–7]. All three are membrane-bound serine proteases that are able to significantly increase ENaC-mediated sodium transport by increasing the open probability (Po) of single channels [6–8] and/or activating a population of near-silent ENaC channels at the plasma membrane [9]. *In vivo* studies conducted on different mouse models for CAP1/Prss8 have shown that this protease is a regulator of ENaC in several epithelia where the two proteins are co-expressed. In lung, absence of CAP1/Prss8 leads to impaired lung fluid clearance mediated by ENaC, and to altered-adrenergic response which may impact on the resolution of pulmonary oedema after lung injury [10–12]. Colon-specific deletion of CAP1/Prss8 resulted in decreased amiloride-sensitive rectal potential difference (PD) upon either regular or low salt diet [13]. Decreased rectal PD was also observed in two spontaneous CAP1/Prss8 mutants, in *frizzy* mice harbouring a V170D transversion, and in frCR rats, that carry a G54-P57 deletion [14–17].

CAP2/Tmprss4, previously termed Tmprss3 [18] belongs to subfamily A of the S1 chymotrypsin family. Proteases of this family are characterized by the presence of a catalytic triad, composed of one histidine (H), one aspartate (D) and a serine (S), forming together a catalytic pocket that enables hydrolysis of target peptide bonds. CAP2/Tmprss4 is a type II transmembrane serine protease, and harbours a N-terminal transmembrane domain, one low-density lipoprotein (LDL) class A domain, one scavenger receptor cysteine-rich (SRCR) domain, the protease domain, and a short C-terminal tail [19,20]. While the physiological role of CAP2/Tmprss4 is largely unknown due to lack of a knockout model, CAP2/Tmprss4 was identified as involved in pathologies such as cancer, influenza infections and neurological disorders [21–23].

Experiments in Xenopus oocytes strongly supported the hypothesis that CAP2/Tmprss4 activates ENaC-mediated sodium current by cleaving the Scnn1g subunit at position R138 [24], previously identified as furin-consensus cleavage site [25,26], although the significance for final ENaC activation is still under debate [27].

In the present study, we aimed to investigate the *in vivo* physiological function of CAP2/Tmprss4 using constitutive knock-out mice. Our data indicate that ENaC-mediated sodium reabsorption is not regulated by CAP2/Tmprss4 arguing for a redundant protease network regulating sodium homeostasis.

## Material and Methods

### Animals and ethics statement

All experimental procedures and animal maintenance followed Swiss federal guidelines. This study has been reviewed and approved (authorization no. 1003.7 to EH) by the “Service de la consommation et des affaires vétérinaires” (SCAV) of the canton of Vaud, Switzerland. Animals were anaesthetized by intraperitoneal injection of 10μl per gram of body weight with a solution containing 10% of Rompun (Bayer) and 10% Ketanarkon (Streuli Pharma) diluted in water. If necessary, animals were sacrificed by cervical dislocation and bleeding. Animals were housed in rooms with controlled temperature and humidity levels and a 12h/12h light/dark cycle, and had free access to food and drinking water. Age-matched homozygous mutant (CAP2/*Tmprss4* ^Δ*/*Δ^, Δ/Δ, knockout, KO), heterozygous mutant (CAP2/*Tmprss4* ^Δ*/+*^, Δ/+, HET), and CAP2/*Tmprss4* wildtype (CAP2/*Tmprss4* ^*+/+*^, +/+, WT) littermates were obtained by interbreeding mice heterozygous mutant for the CAP2/*Tmprss4* ^Δ*/+*^ allele. Genotyping of the 350bp floxed, 500bp knockout and 250bp wildtype allele was performed by PCR on genomic DNA using following primers [5’sense: s3, 5’-GGTCAGATGTAAAAGGTAGAC-3’; VR anti-sense: as3, 5’-CACACCAGCCCTGAATCATC-3’; and 3’-anti-sense: as2 5’-GCTAGGTTCCTTGTTCCTG-3’. PCR amplification was performed for 36 cycles for 1’ at 95°C, 1’ at 56°C and 1’ at 72°C. PCR products were visualized by ethidium bromide staining and run by electrophoresis on 2% agarose gel. Male and female animals (mice homozygous for *Ren-1* ^*c*^), if not stated elsewise, were used at the age of 3 to 6 months and fed with standard (0.17%) Na^+^ diet (ssniff, Spezialdiäten GmbH, Germany).

### Generation of conditional and null mutant CAP2/Tmprss4 mice

To construct the CAP2/*Tmprss4* replacement-type targeting vector, a 14kb genomic DNA contig (strain 129S5/SvEvBrd) spanning exon 6–13 was cloned into pREC-1 vector containing a HSV-TK cassette. A *loxP* site was inserted into the *Bst*EII site upstream of exon 8 resulting in a 4.2kb 5’ homologous region containing exons 6 and 7 and the 1.8kb vital region harbouring exons 8 (histidine, H243) and 9 (aspartate, D288) of the catalytic triad. A 1.5kb *Bam*HI/*Pvu*I FRT-neo-FRT-lox cassette (pAT-FRT-K13; [28]) was introduced into the *Spe*I site, generating the 3.4kb *Spe*I/*Eco*RI 3’ homologous region containing exons 10–13. The targeting vector was linearized with *Sal*I and electroporated into mouse embryonic stem (ES) cells (129S5/SvEvBrd) [29]. Briefly, G418- and ganciclovir-resistant colonies were expanded and screened by PCR using following primers: 3’ recombination: sense 5’-GGACATTGCCCTTGTTAAGCTG-3’ or sense: s1, 5’-TCGCCTTCTTGACGAGTTCTTC-3’ combined with antisense: as1, 5’-GTTTGTCATTGGTGCCGTGTG-3’. Targeted clones were further confirmed by Southern blot analysis using a 5’ external probe (523bp *Nde*I/*Pst*I fragment) revealing 7.5kb wildtype and 9.6kb mutant (*loxneo*) alleles on *Spe*I/*Nhe*I-digested genomic DNA, and using a 3’-probe (530bp *Sph*I/*Sac*I fragment) detecting 7.5kb wildtype and 8.9kb mutant (*loxneo*) alleles, on *Bam*HI-digested DNA as well as an internal probe (PCR-amplified neomycin fragment) which revealed a 4.7kb mutant band on *Eco*RI-digested genomic DNA. Following deletion of the neomycin cassette, the 5’ probe detected a 7.5kb (wildtype or floxed), a 9.6kb (*loxneo*) or a 5.6kb (knockout) fragment on *Spe*I/*Nhe*I-digested genomic DNA. Position of *lox*P sites was verified by PCR with *lox*P-specific primers (details available on request).

Correctly targeted cells (clone #1) were injected into C57BL/6N blastocysts and germline chimeras were obtained. Breeding of CAP2/*Tmprss4* ^*loxneo/loxneo*^ mice with Flp mice [30] allowed the excision of the neomycin cassette to generate mice carrying the CAP2/*Tmprss4* ^*lox*^ (CAP2/*Tmprss4* Lox) allele. Breeding with nestin-Cre mice [31] allowed to generate mice carrying the Δ (CAP2/*Tmprss4* ^Δ^ CAP2/Tmprss4 KO, knockout, Tmprss4^tm1.1Hum^) allele.

### RNA extraction and qRT-PCR

Organs were frozen in liquid nitrogen and stored at -80°C. Tissues were homogenized using TissueLyser (Qiagen, Valencia, CA), and mRNA was isolated using Qiagen RNeasy Mini Kit (Basel, Switzerland) according to the manufacturer’s instructions. cDNA synthesis was performed using 1.5μg of mRNA and reverse transcribed using PrimeScript RT reagent kit according to the manufacturer’s instructions (Takara Bio Inc Japan). Real-time PCR was performed using TaqMan Universal PCR Master Mix (Applied Biosystems) for CAP1/*Prss8*, CAP3/*ST-14*, *Scnn1a*, *Scnn1b*, *Scnn1g*, and furin, or Power SYBRgreen PCR Master Mix (Applied Biosystems) for CAP2/*Tmprss4*, and run using Applied Biosystems 7500 Fast (Carlsbad, CA). Each measurement was performed as duplicate. Quantification of fluorescence was normalized to β*-actin* for TaqMan reagents, and to mouse *Gapdh* for SYBRgreen reagents. Primer and probe sequences for CAP1/*Prss8*, CAP3/*ST-14*, *Scnn1a*, *Scnn1b* and *Scnn1g* have been described previously [13]. The primer sequences used for CAP2/*Tmprss4* were: 5’-CTGCCTTGACTGTGGAAAG-3’ and 5’-GCTGCTTGTTGTACTGGATG-3’, and for furin: 5’-GCCGGAAAGTGAGCCATTC-3’, 5’-GGGTTCCACCAGGATTTCAA-3’ and 5’-FAM-TGCCATGGTGGCTCTGGCCC-BHQ1-3’.

### SDS-PAGE and Western blot analysis

30μg of proteins were separated by SDS-PAGE on 10% acrylamide gels, and proteins were electrically transferred to PolyScreen PVDF hybridization transfer membranes (Perkin Elmer, Boston, MA). Membranes were incubated overnight at 4°C with primary rabbit antibody for Scnn1a (1:500), Scnn1b and Scnn1g (1:1000) [32], CAP2/Tmprss4 [33] (1:200) and β-actin (1:1000, Sigma-Aldrich) and for 1 hour with donkey anti-rabbit IgG HRP-conjugated secondary antibody (1:10000, Amersham, Burkinghampshire, UK) (all antibodies in TBS-Tween 1% and dried milk 2%). The signal was revealed using SuperSignal West Dura detection system (Pierce, Rockford, IL) and quantified using ImageStudio^TM^ Lite program (LI-COR). Kidney extracts from inducible renal tubule-specific Scnn1a KO mice, generated by interbreeding of *Scnn1a* ^*lox/lox*^ mice [34] and *Pax8*::*rtTA/LC1* mice [35], were used as control for Scnn1a-specific signals on Western blot (control non-doxycycline-induced animals [Ctrl WT], control doxycycline-induced animals [Ctrl KO]). The same strategy was applied for Scnn1b- and Scnn1g-specific bands [36]. The specificity of the primary antibody for CAP2/Tmprss4 has been described previously and extensively tested *in vitro* using the *Xenopus* oocyte expression system [33], and corroborated using protein extracts from CAP2/*Tmprss4* knock-out mice that were used as control.

### Histological analyses

Organs were fixed in 4% paraformaldehyde and processed for paraffin embedding. Following organs were taken for histological analyses: skin, kidney, colon, lung, heart, brain, eye, tongue, stomach, small intestine, spleen, spine, femur, testis, uterus, thymus, salivary gland, pancreas, and adrenal gland. 3μm sagittal sections were cut, prepared and stained with eosin and hematoxylin as previously described [17]. Sections were visualized by optical microscopy (Axioplan, Carl Zeiss Microscopy, Jena, Germany) and pictures were taken using an AxioCam HR microscope (Carl Zeiss Microscopy, Jena, Germany).

### Measurement of physiological parameters

Mice were kept in standard cages with free access to food and water and fed with regular sodium (RS: 0.17% Na^+^) or sodium-deficient diet (<0.01% Na^+^) (ssniff, Spezialdiäten GmbH, Germany) for 21 consecutive days. At the end of the experiment, blood samples were collected. Plasma aldosterone levels were measured according to standard procedures using radioimmunoassay (RIA) (Coat-A-Count RIA kit, Siemens Medical Solutions Diagnostics, Ballerup, Denmark) [37]. Samples with values > 1200 pg/ml were further diluted using a serum pool with low aldosterone concentration (<50 pg/ml). Aldosterone concentration is indicated as pg/ml. Plasma electrolytes were analyzed using an Instrumentation Laboratory 943 Electrolyte Analyzer (UK).

### Amiloride-sensitive rectal transepithelial potential difference measurements

Amiloride-sensitive transepithelial rectal potential difference (ΔPD) measurements were performed as described [38,17]. Briefly, amiloride-sensitive rectal ΔPD was measured in the morning and in the afternoon on two days the same week in anaesthetized animals. Rectal PD was monitored by a VCC600 electrometer (Physiologic instruments, San Diego, CA, USA) connected to a chart recorder. After stabilization of PD, saline solution was injected through the first barrel as control procedure and PD was recorded. Saline solution containing 25μmol/l amiloride was injected through the second barrel and PD was recorded. Potential difference was recorded before and after addition of amiloride as amiloride-sensitive ΔPD.

### Statistical analysis

Results are presented as mean ± SEM. Throughout the study, and if not otherwise stated, data were analyzed by one-way ANOVA. *P* < 0.05 was considered statistically significant.

## Results

### Generation of CAP2/Tmprss4 constitutive knockout mice

**Fig 1:**
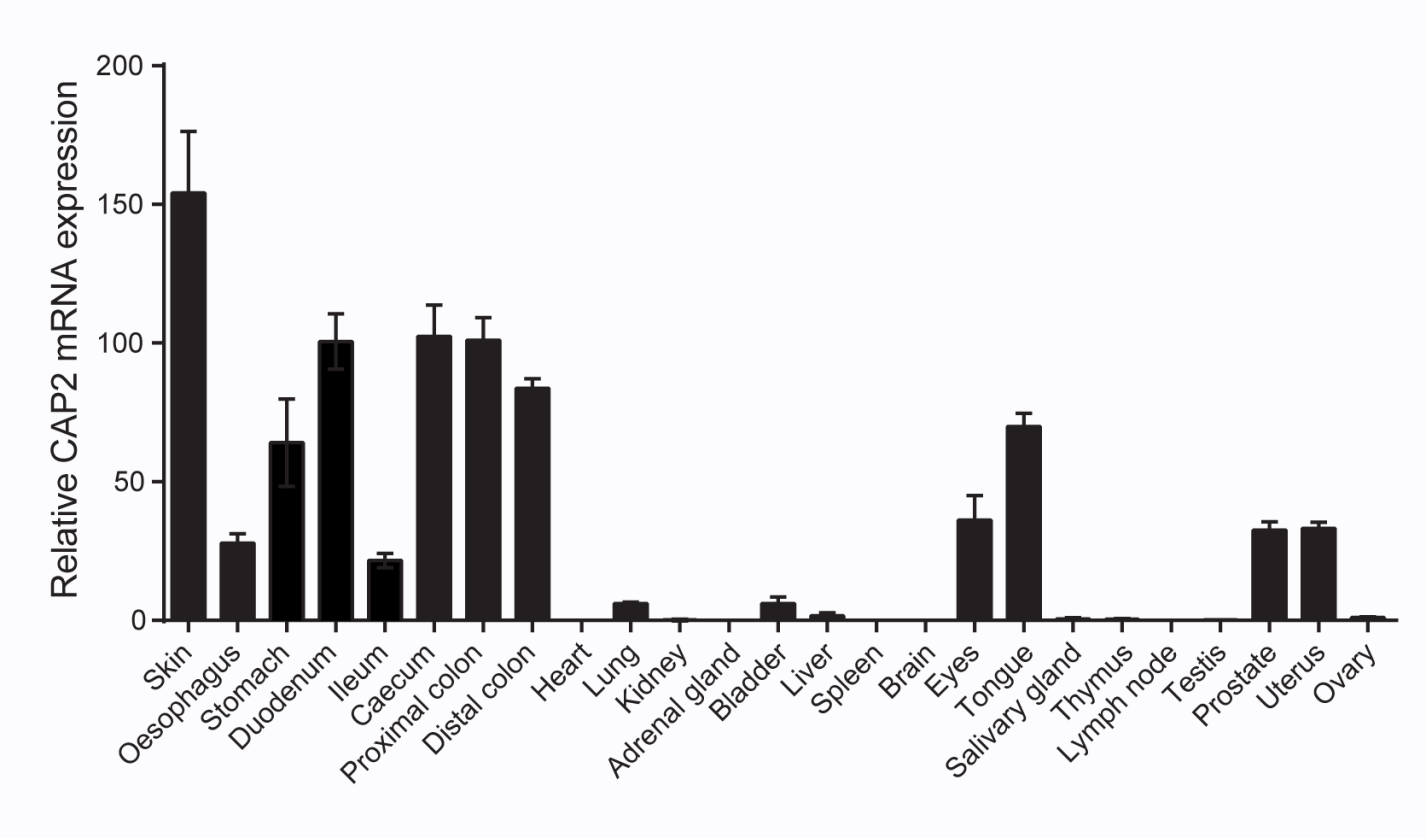
Distribution of wildtype CAP2/*Tmprss4* mRNA transcript expression. CAP2/*Tmprss4* mRNA expression profile in WT mice (n = 4) in various organs as indicated; individual values were normalized to β-actin.

**Fig 2:**
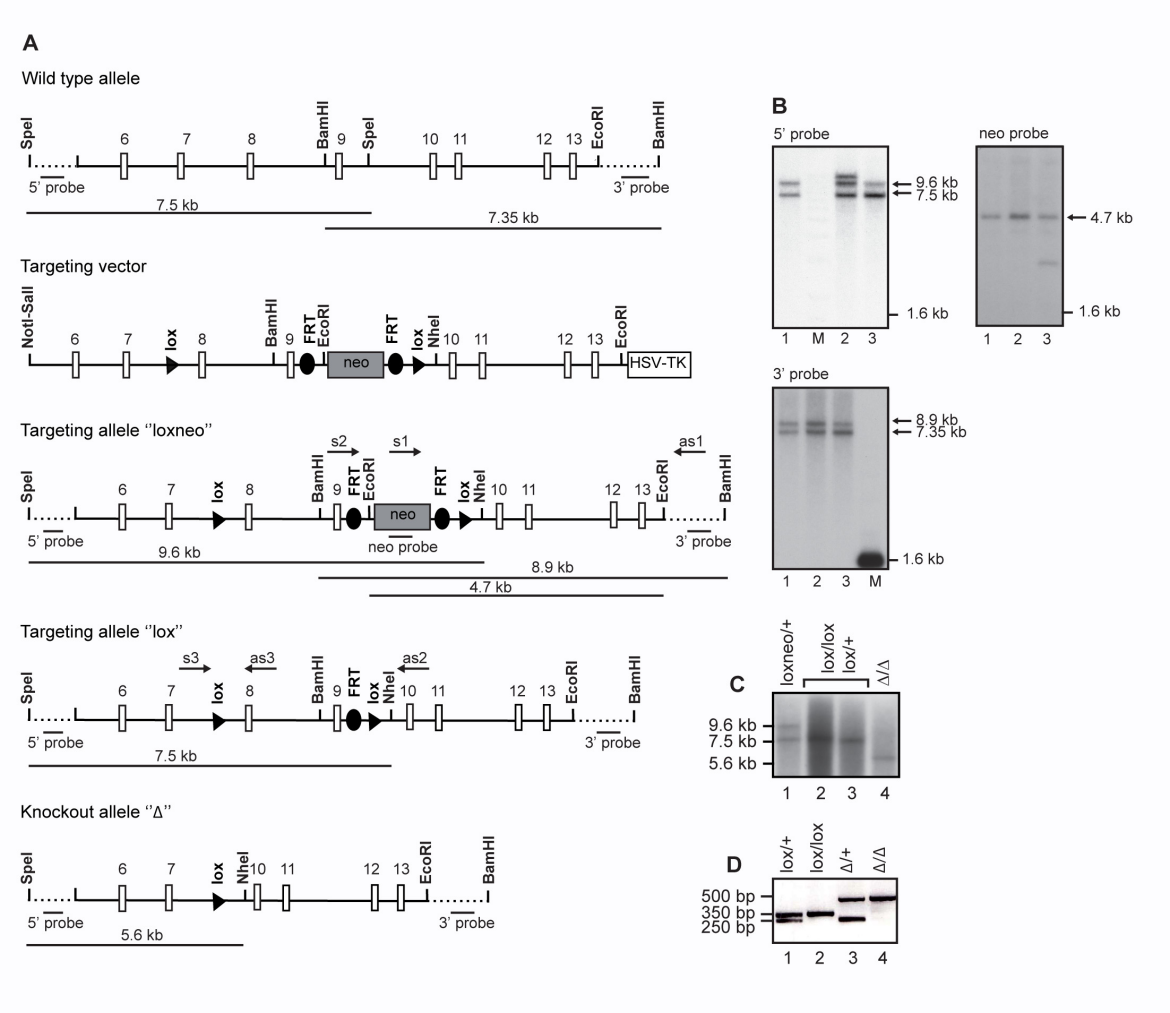
Inactivation of the CAP2/*Tmprss4* gene locus. **(A)** Scheme of the wild-type allele, the targeting vector, and the targeted CAP2/*Tmprss4* ^*loxneo*^ allele following homologous recombination, and the CAP2/*Tmprss4* ^*lox*^ and the CAP2/*Tmprss4* ^Δ^ allele following breeding with Flp- and Cre-deleter mice, respectively. Relevant restriction enzymes for cloning and diagnosis of targeted ES cell clones are shown. Exons 8 and 9 and the neomycin cassette (flanked by *frt* sites) are flanked by *lox*P sites. 5’ and 3’ probes as well as PCR primers used for ES cell screening and mouse genotyping are indicated. (**B**) Southern blot analyses of targeted ES cell clones using the external 5’probe (upper left panel) following digestion with *Spe*I and *Nhe*I, the neo probe (upper right panel) following *Eco*RI digestion, and the external 3’probe following digestion with *Bam*H1; note that clone #2 and #3 harbour additional recombination and integration events as evidenced by Southern blot analyses using the 5’ and neo probe, respectively. (**C**) Southern blot analysis of CAP2/*Tmprss4* ^*loxneo/+*^, CAP2/*Tmprss4* ^*lox/lox*^ and/or CAP2/*Tmprss4* ^*lox/+*^ and CAP2/*Tmprss* ^Δ*/*Δ^ mice using the 5’ probe following *Spe*I/*Nhe*I digestion. (**D**) PCR-based genotyping of mice harbouring the wild type (*+*, 250bp, lane 1 and 3), *lox* alleles (*lox*, 350bp, lane 2) and knockout alleles (Δ, 500bp, lane 3 and 4).

CAP2/Tmprss4, as analysed by quantitative RT-PCR analysis, shows high expression in epithelia like skin and whole digestive tract including duodenum and distal colon, moderate expression in eye, prostate and uterus, low expression in lung, bladder and liver and no detectable expression in whole organs such as heart, kidney, and testis (**Fig 1**). Homologous recombination in mouse embryonic stem (ES) cells was performed to position loxP sides around exons 8 and 9 of the CAP2/*Tmprss4* gene locus containing the histidine and the aspartate of the catalytic triad (**Fig 2A**). Southern blot analyses confirmed correct targeting of ES cell clone #1, which was chosen to generate germline chimeras (**Fig 2B)**. *CAP2/Tmprss4* ^*loxneo/+*^ mice were mated with Cre- or Flp-deleter mouse strains [31,30], and floxed CAP2/*Tmprss4* (*CAP2/Tmprss4* ^*lox/+*^, *CAP2/Tmprss4* ^*loxlox*^), CAP2/*Tmprss4* heterozygous mutant (*CAP2/Tmprss4* ^Δ*/+*^) and knockout (*CAP2/Tmprss4* ^Δ*/*Δ^) mice were obtained as evidenced by Southern blot (**Fig 2C**) and DNA-based PCR analyses (**Fig 2D**).

### CAP2/Tmprss4 knockout mice do not show an obvious phenotype

**Fig 3:**
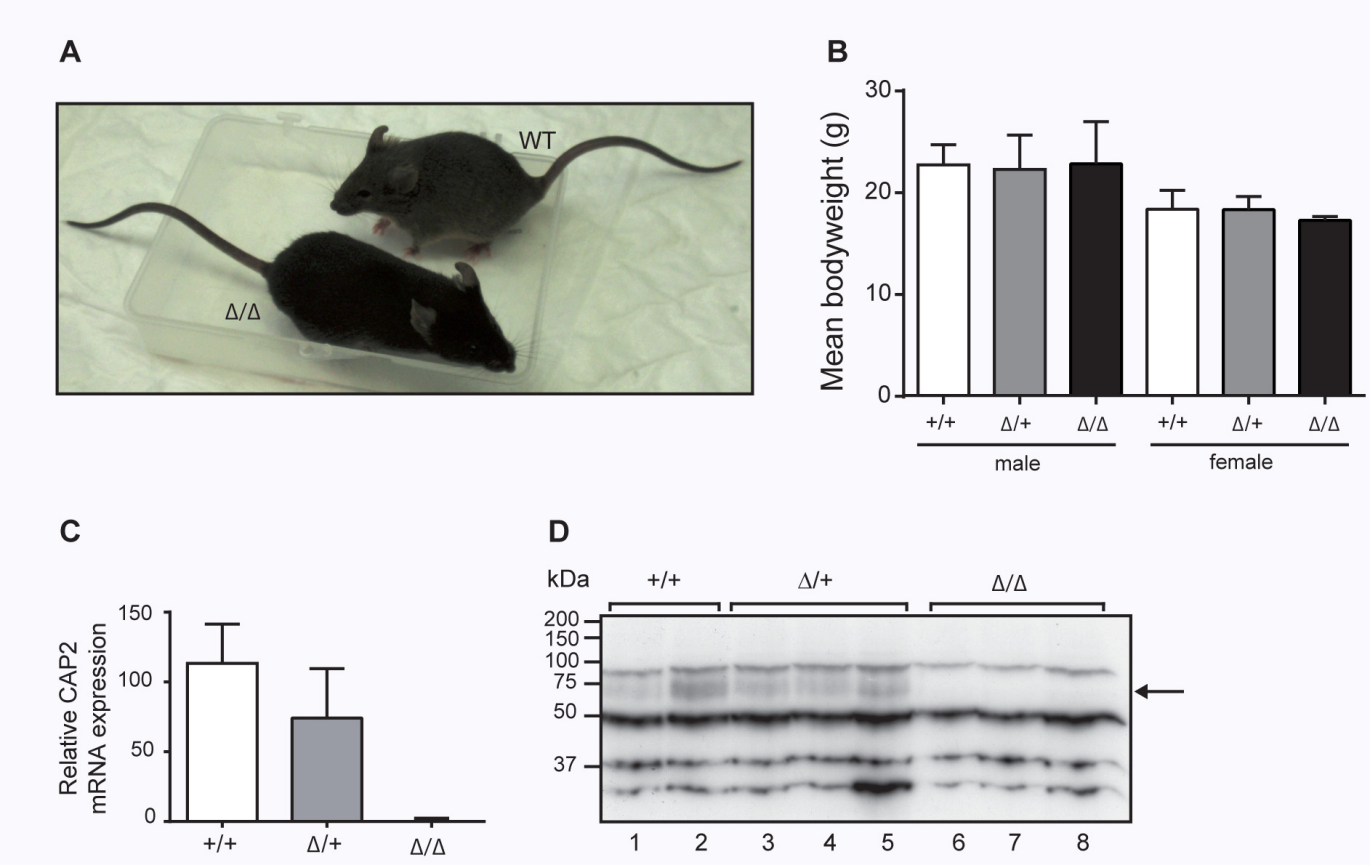
Phenotype of CAP2/*Tmprss4*-deficient mice. **(A)** Representative pictures of 3 months old (male) CAP2/*Tmprss4* wildtype (WT) and CAP2/*Tmprss4* knockout (KO) littermates. (**B**) Mean body weight (g) of 3-month-old male and female wildtype (WT, n = 6), heterozygous mutant (HET, n = 11 and n = 9, respectively), and knockout (KO, n = 6 and n = 5, respectively) mice. (**C**) Relative CAP2/*Tmprss4* mRNA transcript expression in colon from CAP2/*Tmprss4* ^*WT*^, CAP2/*Tmprss4* ^*HET*^ and CAP2/*Tmprss4* ^*KO*^ mice (n = 6 mice per group); β-actin is used as internal control. (**D**) Representative immunoblot showing the presence of a 70kDa CAP2/Tmprss4-specific band in colon extracts from CAP2/*Tmprss4* ^*WT*^ (lane 1 and 2), CAP2/*Tmprss4* ^HET^ (lane 3–5) mice and absence in CAP2/*Tmprss4* ^*KO*^ (lane 6–8) mice; arrow indicates the size of the expected but absent CAP2/Tmprss4-specific band in knockouts.

**Fig 4:**
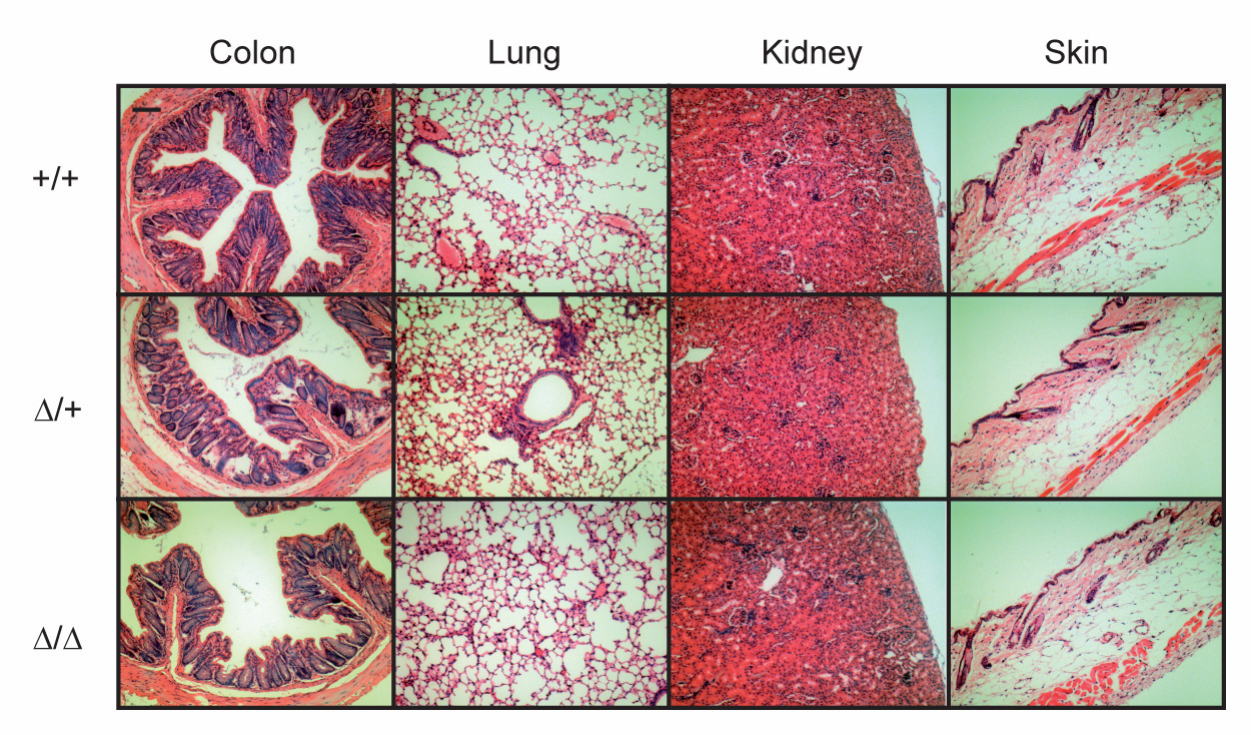
Histopathological analysis in ENaC-expressing organs from CAP2/*Tmprss4* knockout mice. Representative H&E stained section of colon, lung, kidney and skin from CAP2/*Tmprss4* wildtype (WT), heterozygous mutant (HET) and knockout (KO) mice; n = 2 females and 2 males for each group and genotype; bar indicates 100μm.

Following interbreeding of heterozygotes, *CAP2/Tmprss4* wildtype (*CAP2/Tmprss4* ^*+/+*^) heterozygous mutant (*CAP2/Tmprss4* ^Δ*/+*^) and homozygous mutant (*CAP2/Tmprss4* ^Δ*/*Δ^) mice were born according to Mendelian ratio (272 pups: +/+, n = 92; Δ/+, n = 131; Δ/Δ, n = 49; *P* <0.1). CAP2/*Tmprss4* knockout mice appeared healthy and were not affected in body weight (**Fig 3A and 3B**). CAP2/*Tmprss4* knockout mice completely lacked mRNA transcript and protein expression as evidenced by qRT-PCR and Western blot analysis, while heterozygous *CAP2/Tmprss4* mice showed intermediate expression levels (**Fig 3C and 3D**). Histopathology of skin, kidney, colon and lung from knockout mice did not reveal any deviation from wildtype or heterozygous mice (**Fig 4**). Analysis of 16 additional organs revealed no differences either (**data not shown**).

### CAP2/Tmprss4 deletion does not affect ENaC expression and activity

**Fig 5:**
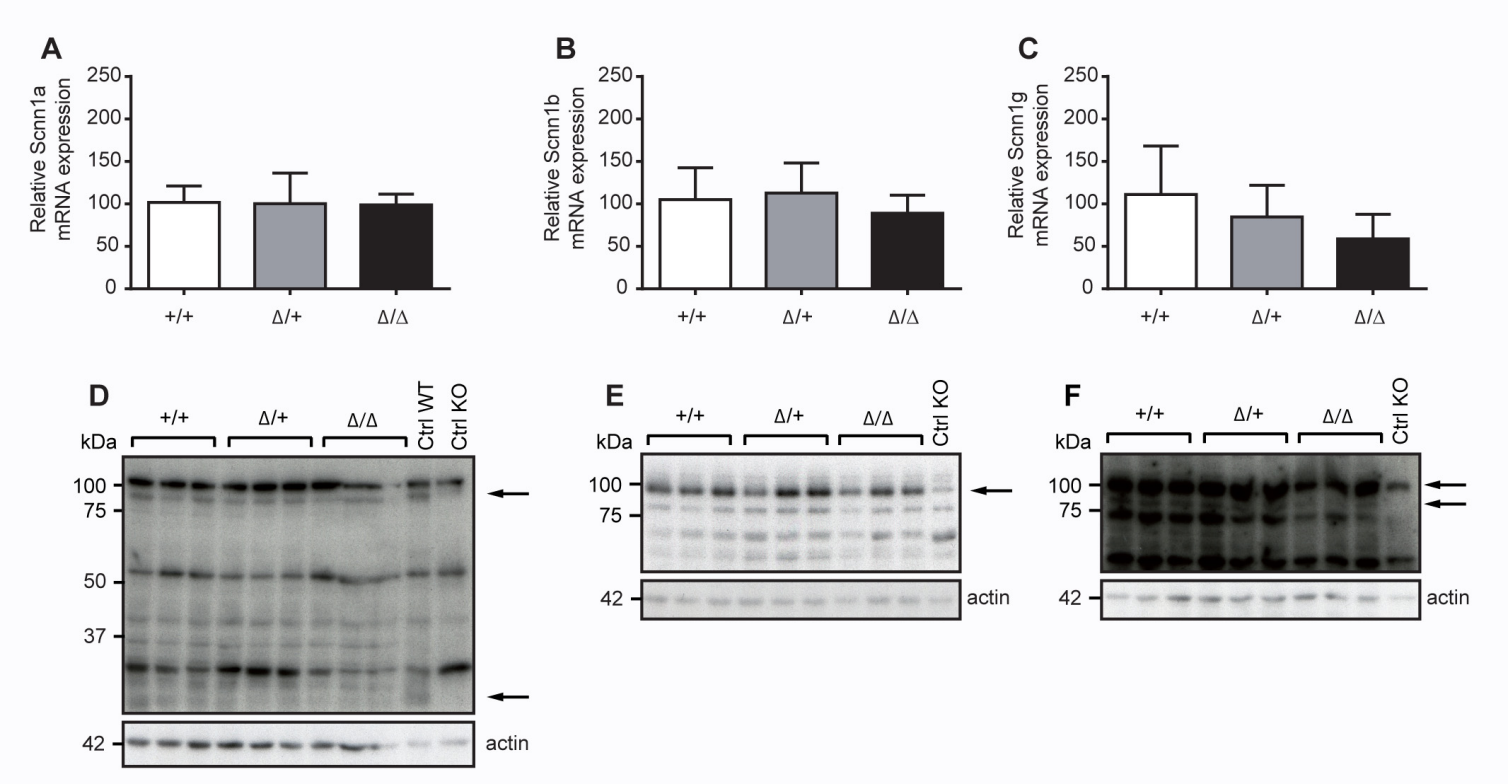
ENaC mRNA transcript and protein expression in kidneys from CAP2/*Tmprss4* wildtype (WT), heterozygous mutant (HET) and knockout (KO) mice under regular sodium diet. (**A-C**) Relative mRNA transcript and (**D-F**) ENaC and β-actin protein expression in kidneys of (**A**) *Scnn1a* in CAP2/*Tmprss4* wildtype (WT, n = 6), heterozygous mutant (HET, n = 7) and knockout (KO, n = 5) mice, (**B**) *Scnn1b* in CAP2/*Tmprss4* wildtype (WT, n = 6), heterozygous mutant (HET, n = 7) and knockout (KO, n = 5) mice, and (**C)** *Scnn1g* in CAP2/*Tmprss4* wildtype (WT, n = 6), heterozygous mutant (HET, n = 5) and knockout (KO, n = 5) mice; β-actin was used as internal control. Representative immunoblots of (**D)** Scnn1a, (**E**) Scnn1b and (**F**) Scnn1g and its corresponding β-actin protein expression in CAP2/*Tmprss4* wildtype (WT), heterozygous mutant (HET) and knockout (KO) mice; kidney extracts from Scnn1 wildtype (WT) and knockout (KO) mice were used as positive and negative control respectively; arrows indicate the full-length and the corresponding cleaved ENaC fragments.

Since channel-activating proteases like CAP2/Tmprss4 are supposed to activate the amiloride-sensitive epithelial sodium channel, thereby possibly affecting the whole net sodium balance, we analysed furthermore renal mRNA transcript expression levels of ENaC subunits in CAP2/*Tmprss4* knockout mice. Here, we could not find any changes between *Scnn1a*, *Scnn1b* and *Scnn1g* expression levels (**Fig 5A, 5B and 5C**). When we analysed ENaC subunit protein expression levels, not only the full-length Scnn1a, Scnn1b or Scnn1g proteins were equally expressed among the different CAP2/Tmprss4 genotypes, but cleaved Scnn1a (32kDa) and Scnn1g (70kDa) ENaC proteins were equally present and expressed (**Fig 5D, 5E and 5F; data not shown**). This also coincides with plasma sodium and potassium, and plasma aldosterone levels that were not significantly different among the genotypes upon regular sodium diet (**Table 1**) indicating that ENaC expression and activity is not affected in kidney. Furthermore, we could not observe any functional redundancy in renal mRNA transcript expression levels among CAP1/*Prss8* and CAP3/*ST-14* (**Table 1**).

**Table 1:** Physiological parameters under regular salt diet or sodium-deficient diet.

**Parameters**	**Regular salt diet**		
	**WT**	Δ**/+**	Δ**/**Δ
n	6	7	5
Body weight (g)	22.70±0.77	22.46±1.59	20.49±1.77
Plasma aldosterone (pg/ml)	358.5±156.8	353.9±126.3	586.6±304.4
Plasma sodium (mmol)	143.15±1.43	145.18±0.66	143.65±0.60
Plasma potassium (mmol)	5.37±0.28	5.32±0.22	5.32±0.11
CAP1, relative mRNA expression (% of control)	100±18.37	97.96±14.78	99.44±17.10
CAP3, relative mRNA expression (% of control)	100±10.16	84.15±6.70	84.80±5.13
Furin, relative mRNA expression (% of control)	n.d.	n.d.	n.d.
	**Sodium-deficient diet**		
	**WT**	Δ**/+**	Δ**/**Δ
n	5	5	4
Body weight (g)	25.75±1.36	25.58±1.48	23.44±2.44
Plasma aldosterone (pg/ml)	803.3±203.6	474.3±157.9	723.7±300.6
Plasma sodium (mmol)	152.20±1.83	155.94±1.97	156.28±3.34
Plasma potassium (mmol)	4.55±0.31	4.79±0.14	4.31±0.29
CAP1, relative mRNA expression (% of control)	100±9.96	119.09±10.06	72.11±4.56
CAP3, relative mRNA expression (% of control)	100±4.72	113.10±10.39	74.93±13.08
Furin, relative mRNA expression (% of control)	100±2.97	123.76±17.44	144.04±8.02

Physiological parameters of CAP2/*Tmprss4* wildtype (WT), heterozygous mutant (HET, Δ/+) and knockout (KO, Δ/Δ) mice under regular sodium or sodium-deficient diets. Data are presented as mean ± SEM.

**Fig 6:**
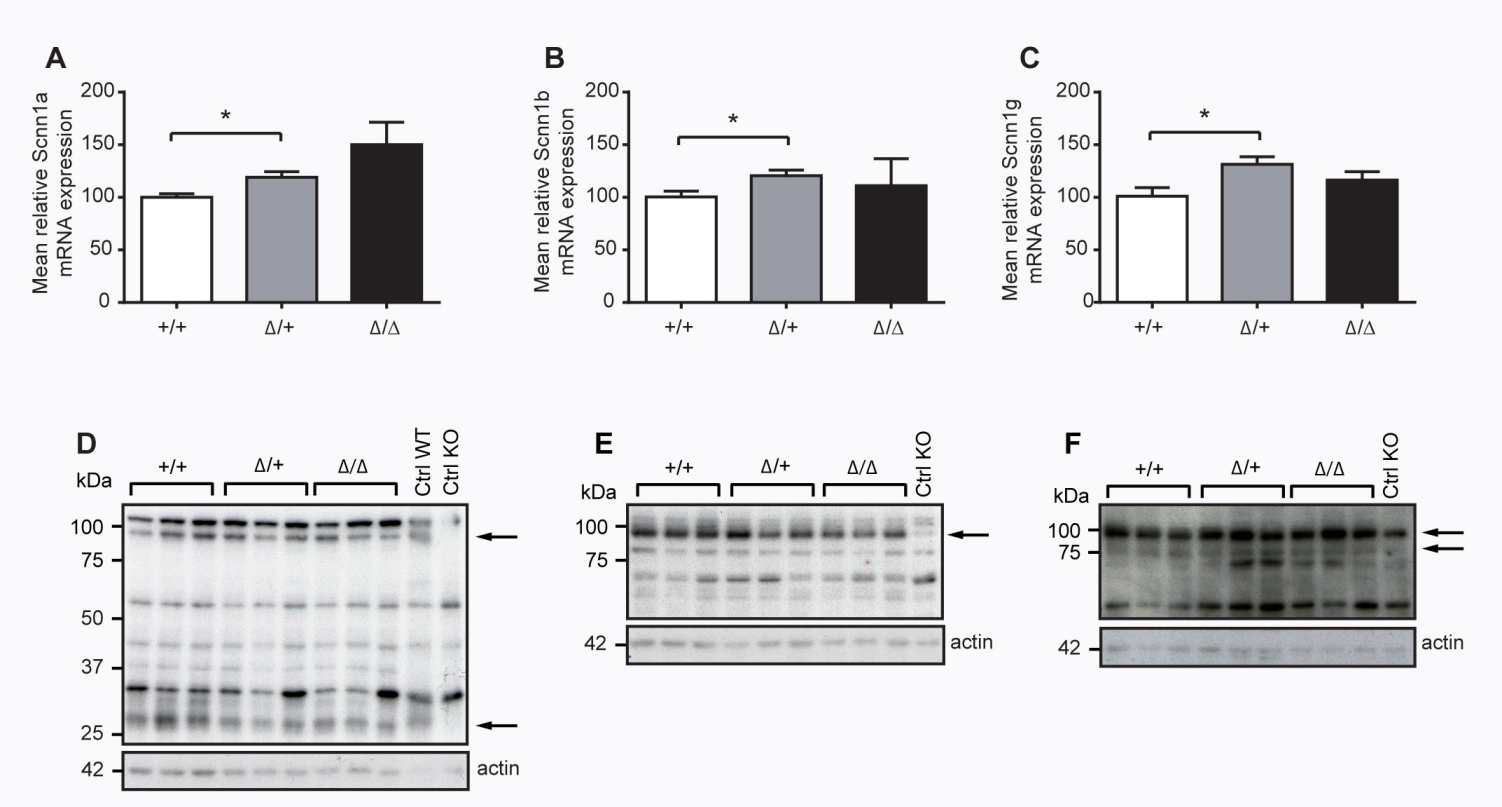
ENaC mRNA transcript and protein expression in kidneys from CAP2/*Tmprss4* wildtype (WT), heterozygous mutant (HET) and knockout (KO) mice under sodium-deficient diet. **(A-C)** Relative mRNA transcript and (**D-F**) protein expression of (**A**) *Scnn1a*, (**B**) *Scnn1b* and (**C)** *Scnn1g* from CAP2/*Tmprss4* wildtype (WT), heterozygous mutant (HET), and knockout (KO) mice; n = 4 for each group and genotype; β-actin was used as internal control. Representative immunoblots of (**D)** Scnn1a, (**E**) Scnn1b and (**F**) Scnn1g and its corresponding β-actin protein expression from CAP2/*Tmprss4* wildtype (WT), heterozygous mutant (HET) and knockout (KO) mice (n = 5 for each group and genotype); kidney extracts from Scnn1 wildtype (WT) and knockout (KO) mice were used as positive and negative control respectively; arrows indicate the full-length and the corresponding cleaved ENaC fragments; * *P*< 0.05).

**Fig 7:**
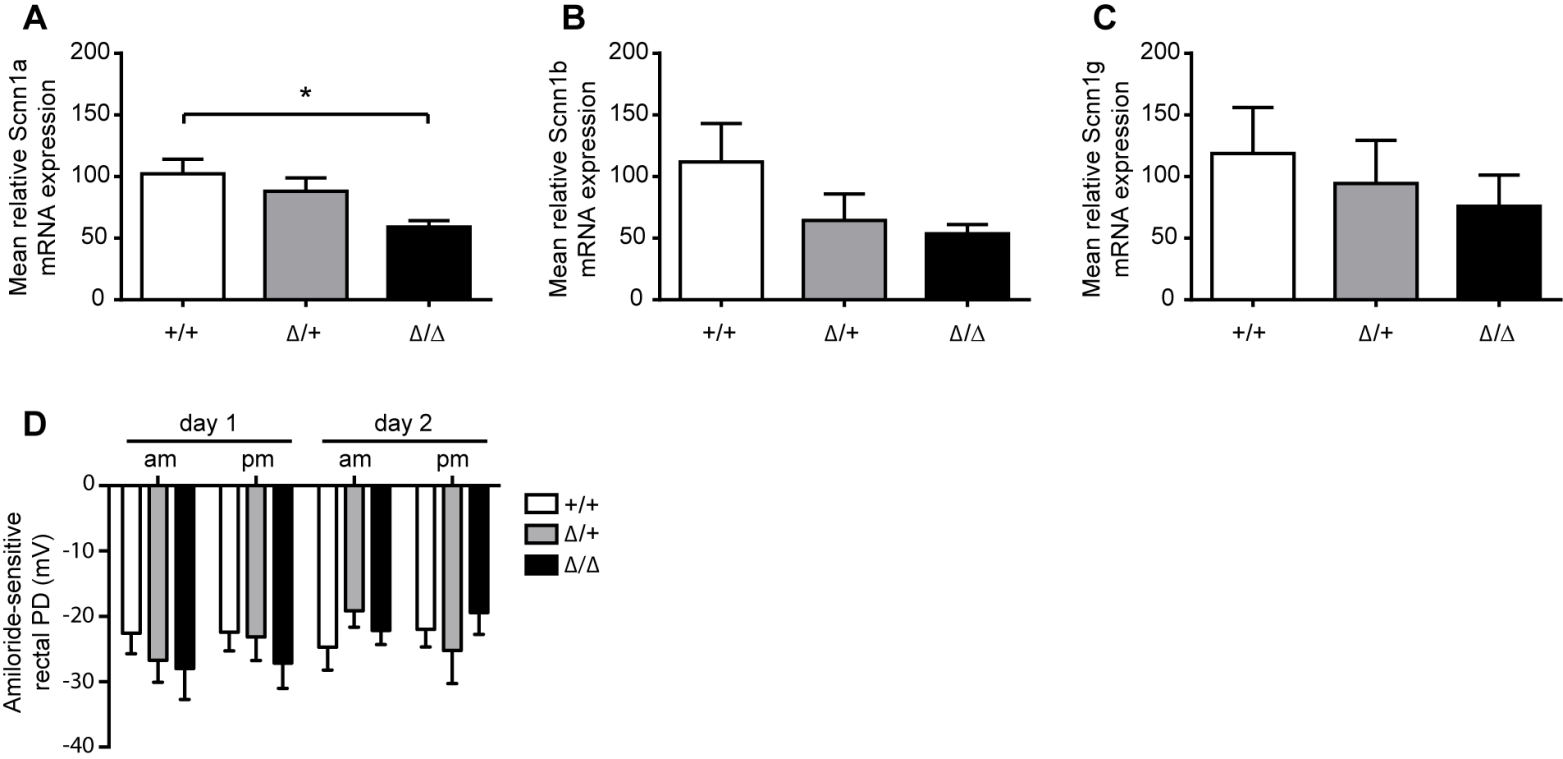
ENaC mRNA transcript expression and activity in colon from CAP2/*Tmprss4* mice under sodium-deficient diet. (**A-C**) Relative mRNA transcript expression of (**A**) *Scnn1a*, (**B**) *Scnn1b* and (**C)** *Scnn1g* from CAP2/*Tmprss4* wildtype (WT, n = 4), heterozygous mutant (HET, n = 5), and knockout (KO, n = 4) mice; *P< 0.05); β-actin was used as internal control. (**D)** Morning and afternoon amiloride-sensitive rectal potential difference (PD) measurements at 10-12am and 4-6pm of two consecutive days in *Tmprss4* wildtype (WT), heterozygous mutant (HET) and knockout (KO) mice; n = 4 for each group and genotype.

When challenging CAP2/*Tmprss4* knockout mice with sodium-deficient diet, we unveiled no alteration in plasma sodium and potassium or plasma aldosterone levels (**Table 1**). Similarly, mRNA transcript expression levels of ENaC subunits did not differ between knockout and wildtype mice, even though we found a significant difference of ENaC subunit expression between wildtype and heterozygous mutant CAP2/*Tmprss4* mice (**Fig 6A, 6B and 6C**). However, this difference could not be confirmed on protein levels and we found no difference in protein expression for full-length Scnn1a, Scnn1b and Scnn1g and cleaved Scnn1a and Scnn1g subunits (**Fig 6D, 6E and 6F; data not shown**). In colon, we found a significantly decreased mRNA transcript expression of *Scnn1a* in CAP2/*Tmprss4* knockout mice under sodium restriction, while mRNA transcript expression of *Scnn1b* and *Scnn1g* did not significantly differ (**Fig 7A, 7B and 7C**). We finally tested *in vivo* ENaC activity upon sodium-deficiency in distal colon and determined the amiloride-sensitive rectal potential difference (ΔPDamil). This did not reveal an effect of CAP2/Tmprss4-deficiency on ENaC activity (**Fig 7D**) demonstrating that CAP2/Tmprss4 is not required for *in vivo* colonic ENaC activity.

## Discussion

In the present study, we generated constitutive knockout mice for CAP2/Tmprss4, targeting exons 8 and 9 that contain two out of three amino acids (histidine and aspartate) of the catalytic triad (**Fig 2**). Disruption of the CAP2/*Tmprss4* gene locus and CAP2/Tmprss4-deficiency was verified at the genomic, mRNA transcript and protein expression level (**Figs. 2 and 3**).

The knockout mice seemed healthy and we detected no obvious effects on embryonic development or after birth (**Fig 3**), in contrary to the phenotype described for CAP2/Tmprss4 knockdown experiments in zebrafish embryos which exhibit severe defects in tissue development and cell differentiation including disturbed skeletal muscle formation, decelerated heartbeat, a degenerated vascular system, and impaired epidermal keratinocytes [39]. This strongly suggests a functional redundancy among serine proteases in the mammalian system, although we could not reveal any upregulation of other channel-activating proteases, such as CAP1/Prss8 (prostasin), CAP3/ST-14 (matriptase) or furin (**Table 1**). Absence of the channel-activating proteases such as CAP1/Prss8 leads to embryonic lethality due to placental failure [40]. Skin-specific conditional knockout of CAP1/Prss8 and a constitutive knockout of CAP3/ST-14 result in early postnatal lethality due to severe impaired skin barrier function [41,42].

Although target substrates of CAP2/Tmprss4 are largely unknown, *in vitro* experiments in *Xenopus* oocytes identified the amiloride-epithelial sodium channel ENaC as potential downstream target [7,24]. *In vitro*, in presence of CAP2/Tmprss4, the open probability (Po) of the amiloride-sensitive ENaC channel is significantly increased and can be blocked by preincubation of *Xenopus* oocytes [7] with aprotinin, an inhibitor of serine proteases [4,6]. Thereby, catalytic activity seems to be required since the mutation of the serine (S385) of the catalytic triad in CAP2/Tmprss4 completely inhibits ENaC activation *in vitro* [33]. Previously, *in vitro* experiments pointed to an implication of CAP2/Tmprss4 in Scnn1g and Scnn1a cleavage, and several putative cleavage sites including the Scnn1g furin (R138) site were reported to significantly reduce ENaC-mediated sodium current [24,43]. Moreover, a recent study confirmed that Scnn1g is processed proteolytically in human kidney [44]. Although CAP2/*Tmprss4* mRNA expression was low in kidney, the protein was previously identified in a mouse cortical collecting duct cell line (mpkCCD_C14_) [7]. mRNA expression was confirmed in the same cell line but could not be detected in whole kidney, suggesting a low and localized expression of CAP2/Tmprss4 in kidney [7]. We thus concentrated on ENaC-expressing organs for histopathology, such as skin, lung, kidney and colon, but could not detect any alterations in CAP2/Tmprss4 knockout or heterozygous mice (**Fig 4**). We expected cleavage changes in Scnn1a and Scnn1g, but did not detect differences of the potentially cleaved 32kDa Scnn1a and 70kDa Scnn1g fragments in CAP2/*Tmprss4* knockout mice, strongly suggesting that *in vivo* cleavage of ENaC is independent of CAP2/Tmprss4 (**Fig 5**).

It has been reported that dietary salt restriction promotes both cleavage and release of an imbedded inhibitory tract from the Scnn1g subunit, that could account for the increased Na^+^ absorption observed in rats on low Na^+^ diet [45–47]. When lowering dietary salt intake, ENaC activity is enhanced upon increased aldosterone secretion to preserve sodium homeostasis [48]. Even though significant increase of mRNA transcript levels for all three ENaC subunits was detected in kidney of heterozygous mutant, but not in knockout CAP2/*Tmprss4* mice, protein levels for full-length and cleaved ENaC subunit forms were unchanged between genotypes. Body weight, plasma aldosterone, sodium and potassium were not changed (**Fig 6 and Table 1**), and no obvious compensation was detected when measuring mRNA levels for CAP1/*Prss8*, CAP3/*ST-14* or furin (**Table 1**).

We cannot exclude that other proteases recently identified as *in vitro* potent ENaC activators, as trypsin IV [49] trypsin I [49] meprin β [50] or cathepsin B [51] might be implicated in *in vivo* ENaC activation. As CAP2/*Tmprss4* mRNA expression level was high in wildtype colon, we investigated whether ENaC mRNA transcript expression could be affected in this organ (**Fig 7**). Although the significant decrease in mRNA transcript expression might be indicative for reduced colonic ENaC activity, monitoring of ENaC activity by amiloride-sensitive PD showed no difference between genotypes (**Fig 7D**). It has been shown that the activity of ENaC is not proportional to the amount of expressed ENaC protein levels, and that *de novo* synthesis of ENaC subunits might play an important role in channel regulation [52]. The detected protein pool (**Figs. 5 and 6**) represents the cytoplasmic as well as the plasma membrane pool of total proteins. Even when ENaC is located at the plasma membrane, the channel can remain silent and not active [9]. The unaltered ENaC activity is thus consistent with the measured physiological parameters such as plasma sodium and potassium, plasma aldosterone and the Scnn1a and Scnn1g protein cleavage pattern that was not altered in the CAP2/*Tmprss4* knockout mice on sodium-deficient diet (**Fig 6 and Table 1**). CAP1/Prss8, in contrary, is implicated in *in vivo* activation of ENaC in colon as mutations in CAP1/Prss8 in *frizzy* mice and *frCR* rats [17], and the colon-specific CAP1/Prss8 knockout led to significant reduced amiloride-sensitive rectal PD and consequently to 2–3 times elevated plasma aldosterone levels to compensate fecal ENaC-mediated sodium loss via the activation of the renin-angiotensin-aldosterone (RAAS) system [13].

Target substrate specificity of CAP2/Tmprss4 under physiological conditions is still largely unknown, and no other target substrate than ENaC has so far been proposed in this area of research. Its implication in pathophysiological processes, however, becomes more evident. CAP2/Tmprss4 was found mutated in a new form of pediatric neurodegenerative disorder, termed Autosomal Recessive Cerebral Atrophy (ARCA), where a point mutation in the gene (c.995C>T) leads to severe CNS degeneration [23]. A role of CAP2/Tmprss4 in influenza virus spreading was proposed, mediated through proteolytic cleavage of the viral protein hemagglutinin (HA), although virus specificity has not been identified so far due to lack of a suitable knockout model [53,22,54]. Upregulation of CAP2/Tmprss4 is observed in various cancer types originating from pancreas, lung, breast, colon and stomach [18,55–60], and was found associated with poor prognosis in patients [59–63].

In conclusion, in this study, we generated and analysed CAP2/Tmprss4 knockout mice and demonstrate that the protease CAP2/Tmprss4 is not required for *in vivo* ENaC-mediated sodium regulation. We propose that these knockout mice can be used to determine the target substrate specificity and its further implication in physiological and pathophysiological processes.
